# Prevalence of pneumonia in early childhood and associated factors in a location with high pneumococcal vaccination coverage

**DOI:** 10.1590/0102-311XEN125424

**Published:** 2025-11-07

**Authors:** Nilvia Soares Aurélio, Isabel Oliveira Bierhals, Andréa Dâmaso Bertoldi, Mariângela Freitas da Silveira, Iná da Silva dos Santos

**Affiliations:** 1 Universidade Federal de Pelotas, Pelotas, Brasil.; 2 Empresa Brasileira de Serviços Hospitalares, Pelotas, Brasil.; 3 Centro de Pesquisas Epidemiológicas, Universidade Federal de Pelotas, Pelotas, Brasil.; 4 Universidade do Extremo Sul Catarinense, Criciúma, Brasil.; 5 Faculdade de Medicina, Universidade Federal de Pelotas, Pelotas, Brasil.

**Keywords:** Pneumonia, Risk Factors, Pneumococcal Vaccine, Children, Pneumonia, Fatores de Risco, Vacinas Pneumocócicas, Crianças, Neumonía, Factores de Riesgo, Vacunas Neumocócicas, Niños

## Abstract

Pneumonia constitutes a leading cause of death in children from low- and middle-income countries. We aimed to describe the prevalence and the factors associated with pneumonia in children aged from 0 to 6 years in a location with high pneumococcal vaccination coverage. The occurrence of at least one episode of pneumonia diagnosed by a physician as reported by the mother was investigated at the 12-, 24-, and 48-month, and 6-year follow-ups of the 2015 Pelotas (Brazil) birth cohort. The independent variables included family and child characteristics at birth, breastfeeding, and vaccinal status. Prevalence ratios (PR) with 95% confidence interval (95%CI) were estimated by unadjusted and multivariable Poisson regressions with robust variance. At the 12-, 24-, 48-month, and 6-year follow-ups 4,014, 4,006, 3,997, and 3,862 children were assessed, respectively. Prevalence from 0 to 6 years equaled 16.7% (95%CI: 15.5-18.0). Within the first, second, 2-4 and 4-6 years of age the prevalence of at least one episode of pneumonia totaled 7.9%, 5.9%, 6.7%, and 3.4%, respectively. Higher maternal parity (adjusted PR = 1.75, 1.61, and 2.0 at the first, second, and 4-6 years, respectively) and prematurity (adjusted PR = 1.39 and 1.49 at the first and second years of life, respectively) constituted the factors most consistently associated with an increased risk of pneumonia. Almost one in every five children aged 6 years had a positive history of pneumonia, mainly in their first year of life. Greater maternal parity was the strongest and most consistent factor associated with a higher prevalence of pneumonia in childhood.

## Resumo

Pneumonia é uma das principais causas de morte entre crianças em países de baixa e média renda. Procuramos descrever a prevalência e os fatores associados à pneumonia em crianças de 0 a 6 anos de idade em um local com alta cobertura vacinal pneumocócica. A ocorrência de pelo menos um episódio de pneumonia diagnosticado por um médico, conforme relatado pela mãe, foi investigada nos acompanhamentos de 12, 24 e 48 meses e de 6 anos da coorte de nascimentos de Pelotas, Rio Grande do Sul, Brasil, de 2015. As variáveis independentes incluíram características da família e da criança ao nascer, amamentação e estado vacinal. Razões de prevalência (RP) com respetivos intervalos de 95% de confiança (IC95%) foram estimadas por meio de regressão de Poisson não ajustada e multivariável com variância robusta. Ao total, 4.014, 4.006, 3.997 e 3.862 crianças foram avaliadas nos acompanhamentos de 12, 24, 48 meses e 6 anos, respectivamente. A prevalência de pelo menos um episódio de pneumonia de 0 a 6 anos de idade foi de 16,7% (IC95%: 15,5-18,0). As prevalências no primeiro, segundo, 2-4 e 4-6 anos foram de 7,9%, 5,9%, 6,7% e 3,4%, respectivamente. Maior paridade materna (RP ajustada de 1,75, 1,61 e 2,0 no primeiro, segundo e 4-6 anos, respectivamente) e prematuridade (RP ajustada de 1,39 e 1,49 no primeiro e segundo ano de idade, respectivamente) foram os fatores mais consistentemente associados ao aumento do risco de pneumonia. Quase uma em cada cinco crianças de 6 anos tinha histórico de pneumonia, principalmente no primeiro ano de vida. Maior paridade materna foi o fator mais forte e consistentemente associado à maior prevalência de pneumonia na infância.

## Resumen

La neumonía es una de las principales causas de mortalidad infantil en países de ingresos bajos y medios. Este estudio describe la prevalencia y los factores asociados con la neumonía en niños de 0 a 6 años de edad, en una localidad con alta cobertura de vacunación antineumocócica. Se investigó la ocurrencia de al menos un episodio de neumonía diagnosticado por un médico, según lo informado por la madre, en los seguimientos realizados a los 12, 24 y 48 meses, y a los 6 años de edad en la cohorte de nacimientos de Pelotas, Rio Grande do Sul, Brasil, de 2015. Las variables independientes incluyeron características familiares e infantiles al nacimiento, estado de lactancia materna y situación vacunal. Se estimaron razones de prevalencia (RP) con intervalo de 95% de confianza (IC95%) mediante regresión de Poisson no ajustada y multivariable con varianza robusta. En los seguimientos a los 12, 24, 48 meses y 6 años, se evaluaron respectivamente 4.014, 4.006, 3.997 y 3.862 niños. La prevalencia acumulada de 0 a 6 años fue del 16,7% (IC95%: 15,5-18,0). En los periodos de 0-1, 1-2, 2-4 y 4-6 años, la prevalencia de al menos un episodio de neumonía fue del 7,9%; 5,9%; 6,7% y 3,4%, respectivamente. Una mayor paridad materna (RP ajustada de 1,75; 1,61 y 2,0 en el primer año, segundo año y periodo de 4-6 años, respectivamente) y la prematuridad (RP ajustada de 1,39 y 1,49 en el primer y segundo año de vida, respectivamente) fueron los factores asociados de manera más consistente con un mayor riesgo de neumonía. Aproximadamente uno de cada cinco niños de 6 años tenía antecedentes de neumonía, principalmente durante el primer año de vida. La mayor paridad materna fue el factor más fuerte y consistente asociado con una mayor prevalencia de neumonía en la infancia.

## Introduction

Acute respiratory infections stand among the main causes of mortality in children ^1^. Although deaths due to pneumonia in this age group are largely preventable, community-acquired pneumonia corresponds to approximately 19% of causes of child death worldwide ^2^. In recent decades, substantial advances occurred in the understanding of the risk factors and the etiology of pneumonia, as well as in the development of standardized case definitions and prevention, with the production of vaccines and specific treatments. Such advances changed the epidemiology, etiology, and mortality of childhood pneumonia (although access to these interventions remains precarious in many areas, with great inequalities between and within countries and regions) ^3^.

Most cases of pneumonia occur in low- and middle-income countries. Brazil stands among the 15 countries with the highest prevalence of pneumonia in children aged < 5 years ^4^. On average, one in every 66 children < 5 years in high-income countries suffers from pneumonia per year when compared to one in every five in low- and middle-income countries ^5^. Estimates to case fatality rates suggest numbers nearly 10 times higher in low- and middle-income countries than in high-income countries ^5^ ^,^ ^6^ ^,^ ^7^.

In 2010, Brazil introduced the 10-valent pneumococcal conjugate vaccine (PCV10) into its routine free Brazilian National Immunization Program (PNI, acronym in Portuguese) via the Brazilian Unified National Health System (SUS, acronym in Portuguese) ^8^. Data on hospitalization for pneumonia in five major Brazilian state capitals indicated that the PCV10 reduced hospitalization rates for pneumonia in children by around 30% in three capitals ^9^ ^,^ ^10^. Pneumonia hospitalization rates in children aged < 4 years (including vaccinated and unvaccinated children) decreased by 12.7% from the pre-PCV10 period (2002-2009) to the two years after its introduction ^11^. Nonetheless, population-based studies to assess prevalence and factors associated with pneumonia after the PCV10 vaccination remain scarce in Brazil. Thus, using data from the 2015 Pelotas (Brazil) birth cohort - the first Pelotas cohort after the introduction of PCV10 and with a high coverage of vaccination ^12^ - this study aimed to describe the prevalence and factors associated with it and reoccurrence of pneumonia in children aged from 0 to 6 years.

## Methods

The 2015 Pelotas birth cohort, a population-based longitudinal study, was carried out with women who lived in the urban area of ​​the municipality of Pelotas, southern Brazil, and who had a confirmed pregnancy and expected birth in 2015 in the municipal maternity wards. Children who were born in Pelotas in 2015 and living in its urban area were eligible for the cohort. Of the 5,610 children who were born in Pelotas in 2015, 4,387 were eligible, of whom 4,275 were enrolled in the cohort.

After the baseline perinatal study (conducted at the hospital of birth), follow-ups occurred at 3, 12, 24, and 48 months and at 6 years of life ^13^. Details about that study can be found elsewhere ^13^ ^,^ ^14^. This study included children with available information on the occurrence of pneumonia that were collected at the 12-month follow-up (pneumonia in the first year of life), 24-month follow-up (pneumonia in the second year of life), and 48-month follow-up (pneumonia from 24 to 48 months), and at the 6-year follow-up (pneumonia from 48 months to 6 years).

### Outcomes

The following outcomes were chosen: the prevalence of at least one episode of pneumonia (as informed by the mothers and diagnosed by medical physicians in the first and second years of life, from the second to the fourth year, from the fourth to the sixth year of life, and throughout childhood - from 0 to 6 years) and the reoccurrence of pneumonia (at least two episodes) from age 0 to 6 years. The information was collected by structured interviews with the aid of questionnaires applied to the mothers/guardians. At the 12-month follow-up, the mothers were requested to answer the question: “Has the child ever had pneumonia?” and at the 24- and 48-month and 6-year follow-ups the question was: “Since the previous follow-up, has the child had pneumonia?”. In all follow-ups, after an affirmative answer, the mothers were asked to answer: “Who told you it was pneumonia?”. Only when the answer was “a medical physician”, the child was recorded as a case of pneumonia.

### Independent variables

Based on the perinatal study, family economic situation, household crowding, maternal characteristics (age, skin color, education, parity, and smoking during pregnancy), paternal education, child’s sex, prematurity, and low birth weight were used in this research. Family economic situation was classified using the Brazilian Economic Classification Criteria ^15^ (A-B, C, and D-E), household crowding was evaluated based on the number of household inhabitants divided by the number of rooms used for sleeping (> 2 or ≤ 2), mother’s age in completed years was classified as < 20, 20-34, and ≥ 35, and self-reported maternal skin color was recorded ^16^ as white, mixed-race, black, yellow, and Indigenous, and later classified into two groups (white and mixed-race/black/other). Maternal and paternal education was categorized into 0-8, 9-11, and ≥ 12 years complete years of study. Parity (total number of living or dead children ever born) was grouped into 1, 2, and ≥ 3.

Children’s sex (male or female), prematurity (< 37 weeks of gestational age), and low birth weight (< 2,500g) information was also used in this study. Information on gestational age was based on the date of the last menstrual period and maternal ultrasound (according to the pregnancy card). Weight was measured at the hospital using the SECA model 376 portable pediatric scale (SECA; https://www.seca.com) by trained research staff.

Based on the 3-month follow-up, exclusive breastfeeding (yes or no) was defined according to the World Health Organization (WHO) criteria ^17^. Breastfeeding, number of people in the household, child daycare center attendance, maternal smoking, and doses of pneumococcal vaccine were obtained at the 12-, 24- and 48-month follow-ups. Breastfeeding was classified as yes or no. The number of people in the household was classified as ≤ 3 and > 3. Attendance to child daycare center was categorized as yes or no. Information on immunization with the pneumococcal vaccine was collected from the vaccination cards that were shown to interviewers or from mothers’ verbal reports - categorized into complete vaccination for age (no or yes), in agreement with the PNI, on the date of information collection ^8^. At 12 months, complete vaccination was defined as two or more doses of the vaccine and at 24 and 48 months, as three doses or more. In each follow-up, maternal smoking was defined as smoking at least one cigarette per day.

### Statistical analyses

All analyses were performed on Stata, version 18.0 (https://www.stata.com). The chi-squared heterogeneity test was used to compare sample subsets comprising participants included in the analyses at each follow-up with the original cohort. Period prevalence of pneumonia was described as proportions (%) with 95% confidence intervals (95%CI). Simple and multivariable Poisson regressions with robust variance were used to investigate factors associated with each outcome. Prevalence ratios (PR) with 95%CI were estimated.

For the multivariable analyses, the independent variables were classified into hierarchical groups. For occurrence of pneumonia in the first year, and from birth to 6 years, as well as for reoccurrence, the variables were classified into three hierarchical levels. The most distal of which included socioeconomic characteristics and maternal and paternal variables collected at baseline. The intermediate level comprised maternal smoking during pregnancy, and the proximal level was composed by variables from the child at birth.

For occurrence of pneumonia after the first year of life, the variables were classified into two more levels, with exclusive breastfeeding at three months at the fourth level and maternal smoking, breastfeeding, number of people in the household, attendance to child daycare center, and vaccinal status for pneumococcal vaccine in the fifth level. The variables from the fifth level were those that were recorded at the baseline of the assessed interval. For instance, for pneumonia at the second year of life, the information collected at 12 months was used.

For each level, variables from the same level were simultaneously introduced in addition to the variables from previous levels with a p-value ≤ 0.20 that were kept in the model as potential confounders.

### Ethical aspects

The baseline evaluation of the 2015 Pelotas (Brazil) birth cohort and all its follow-ups were approved by the Ethics Committee of the Faculty of Physical Education of the Federal University of Pelotas (registration numbers 26746414.5.0000.5313 and 26746414.5.0000.531). An informed consent form was signed by the mothers or guardians before the interviews and exams.

## Results

**Figure 1:**
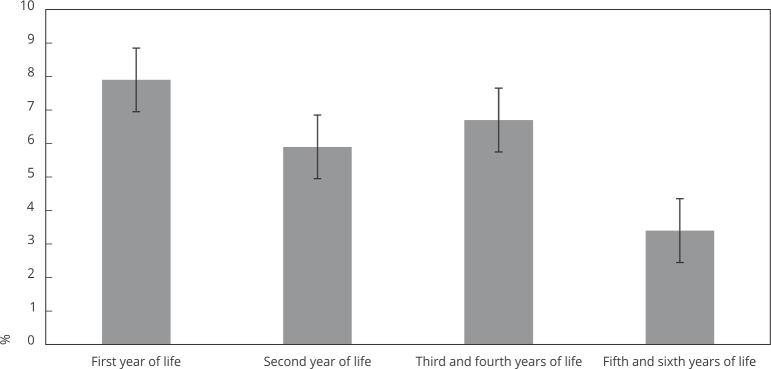
Prevalence of at least one episode of pneumonia in the periods of life from birth to six years of life. The 2015 Pelotas (Brazil) birth cohort.

The 12-, 24-, and 48-month and 6-year follow-ups assessed 4,014, 4,006, 3,997, and 3,862 children, respectively. Table 1 shows the characteristics of the original sample and the participants in each follow-up. No statistical differences were found between the samples and the original cohort. Prevalence of complete pneumococcal vaccination according to age at 12, 24, and 48 months in the analyzed children equaled 95.9%, 84.5%, and 94.1%, respectively. In the first, second, 2-4 and 4-6 years of life the prevalence of at least one episode of pneumonia equaled 7.9% (95%CI: 6.8-8.4), 5.9% (95%CI: 5.2-6.6), 6.7% (95%CI: 6.0-7.5), and 3.4% (95%CI: 2.9-4.0), respectively (Figure 1). The prevalence of at least one episode of pneumonia from 0 to 6 years totaled 16.7% (95%CI: 15.5-18.0). Most children (94.5%) had only endured one episode of pneumonia throughout their childhood, in general at their first year of life. A small proportion of participants (5.5%; 95%CI: 4.8-6.2) reported two or more episodes up to their children’s six years of life.

**Table 1:** Description of the original sample and of participants included in the analysis at each follow-up. The 2015 Pelotas (Brazil) birth cohort.

Characteristics	Original cohort (N = 4,275)	12-month follow-up (N = 4,014)		24-month follow-up (N = 4,006)		48-month follow-up (N = 3,997)		6-year follow-up (N = 3,862)	
	n (%)	n (%)	p-value *	n (%)	p-value *	n (%)	p-value *	n (%)	p-value *
Maternal perinatal									
Family socioeconomic status			0.790		0.926		0.832		0.828
A-B	1,275 (30.9)	1,196 (30.8)		1,194 (30.8)		1,174 (30.4)		1,130 (30.3)	
C	2,047 (49.5)	1,948 (50.2)		1,932 (49.9)		1,941 (50.2)		1,873 (50.1)	
D-E	808 (19.6)	738 (19.0)		745 (19.3)		749 (19.4)		731 (19.6)	
Domestic crowding (> 2 people/bedroom)			0.946		0.982		0.838		0.819
No	1,678 (40.9)	1,621 (40.8)		1,614 (40.9)		1,596 (40.7)		1,541 (40.6)	
Yes	2,425 (59.1)	2,351 (59.2)		2,335 (59.1)		2,329 (59.3)		2,252 (59.4)	
Maternal age (years)			0.997		0.954		0.978		0.913
< 20	623 (14.6)	585 (14.6)		577 (14.4)		581 (14.5)		570 (14.8)	
20-34	3,018 (70.6)	2,837 (70.7)		2,841 (70.9)		2,830 (70.8)		2,731 (70.7)	
≥ 35	633 (14.8)	592 (14.7)		588 (14.7)		586 (14.7)		560 (14.5)	
Maternal education (years)			0.784		0.905		0.697		0.716
0-8	1,486 (34.8)	1,377 (34.3)		1,388 (34.7)		1,388 (34.7)		1,348 (34.9)	
9-11	1,458 (34.1)	1,398 (34.8)		1,384 (34.5)		1,394 (34.9)		1,342 (34.8)	
≥12	1,330 (31.1)	1,238 (31.9)		1,233 (30.8)		1,214 (30.4)		1,171 (30.3)	
Paternal education (years)			0.892		0.883		0.652		0.661
0-8	1,701 (42.3)	1,602 (42.3)		1,598 (42.3)		1,610 (42.8)		1,566 (43.0)	
9-11	1,279 (31.8)	1,219 (32.2)		1,214 (32.2)		1,214 (32.2)		1,163 (31.9)	
≥ 12	1,043 (25.9)	966 (25.5)		961 (25.5)		942 (25.0)		912 (25.1)	
Maternal skin color			0.942		0.866		0.629		0.770
White	3,024 (70.9)	2,843 (70.9)		2,827 (70.7)		2,808 (70.4)		2,720 (70.5)	
Mixed-race/Black/Other	1,244 (29.1)	1,165 (29.1)		1,173 (29.3)		1,183 (29.6)		1,136 (29.5)	
Parity			0.934		0.976		0.983		0.884
1	2,112 (49.4)	1,988 (49.6)		1,988 (49.7)		1,977 (49.5)		1,927 (49.9)	
2	1,321 (30.9)	1,248 (31.1)		1,235 (30.8)		1,239 (31.0)		1,188 (30.8)	
≥ 3	840 (19.7)	776 (19.3)		781 (19.5)		779 (19.5)		745 (19.3)	
Children’s at birth									
Sex			0.943		1.000		0.982		0.929
Male	2,164 (50.6)	2,041 (50.8)		2,027 (50.6)		2,022 (50.6)		1,951 (50.5)	
Female	2,111 (49.4)	1,973 (49.2)		1,979 (49.4)		1,975 (49.4)		1,911 (49.5)	
Prematurity			0.269		0.311		0.282		0.278
No	3,582 (84.4)	3,398 (85.3)		3,388 (85.2)		3,385 (85.2)		3,273 (85.3)	
Yes	663 (15.6)	587 (14.7)		589 (14.8)		586 (14.8)		566 (14.7)	
Low birth weight (< 2,500g)			0.354		0.482		0.482		0.551
No	3,830 (90.0)	3,633 (90.6)		3,620 (90.4)		3,612 (90.4)		3,488 (90.4)	
Yes	428 (10.0)	378 (9.4)		383 (9.6)		382 (9.6)		372 (9.6)	
Breastfeeding pattern at 3 months			0.966		0.978		0.965		0.923
Exclusive	1,834 (44.7)	1,790 (45.1)		1,781 (45.1)		1,767 (45.0)		1,711 (45.1)	
Predominant	305 (7.4)	298 (7.5)		290 (7.3)		291 (7.4)		285 (7.5)	
Partial	999 (24.4)	965 (24.3)		962 (24.4)		962 (24.6)		929 (24.5)	
Weaned	964 (23.5)	918 (23.1)		914 (23.1)		903 (23.0)		866 (22.9)	
Complete pneumococcal vaccination at 3 months **			0.834		0.631		0.811		0.585
No	687 (16.8)	658 (16.6)		645 (16.3)		649 (16.5)		617 (16.3)	
Yes	3,414 (83.3)	3,314 (83.4)		3,303 (83.7)		3,274 (83.5)		3,175 (83.7)	
Complete pneumococcal vaccination at 12 months ***			1.000		1.000		0.818		0.640
No	162 (4.1)	162 (4.1)		157 (4.1)		152 (4.0)		143 (3.9)	
Yes	3,753 (95.9)	3,750 (95.9)		3,651 (95.9)		3,640 (96.0)		3,519 (96.1)	
Complete pneumococcal vaccination at 24 months ^#^					0.951		0.804		0.615
No	624 (15.6)	-		620 (15.5)		599 (15.3)		570 (15.1)	
Yes	3,390 (84.4)	-		3,386 (84.5)		3,310 (84.7)		3,200 (84.9)	
Complete pneumococcal vaccination at 48 months ^#^							0.960		0.506
No	214 (5.9)	-		-		212 (5.9)		191 (5.5)	
Yes	3,414 (94.1)	-		-		3,407 (94.1)		3,265 (94.5)	

* Chi-squared test when compared to the original cohort;

** 1 dose;

*** 3 doses;

^#^ 4 doses.

### Pneumonia in the first year of life

Of the infants whose mothers had ≥ 3 or 2 children, pneumonia in the first year of life was 1.75 and 1.34 times higher, respectively, than among those who were firstborn (PR = 1.75; 95%CI: 1.31-2.34, and PR = 1.34; 95%CI: 1.02-1.75) (Table 2). Boys had a 56% greater risk of pneumonia than girls (PR = 1.56; 95%CI: 1.24-1.96). Prematurely born individuals showed a higher occurrence of pneumonia (PR = 1.39; 95%CI: 1.05-1.83). Lower family socioeconomic status, lower maternal and paternal education, and maternal smoking during pregnancy increased the probability of pneumonia in the first year of life in the unadjusted analysis (which lost its statistical significance after the addition of confounders).

**Table 2:** Factors associated with the prevalence of pneumonia in the first year of life. The 2015 Pelotas (Brazil) birth cohort.

Variables	History of pneumonia					
	Unadjusted			Adjusted		
	PR	95%CI	p-value	PR	95%CI	p-value
Level 1						
Family socioeconomic status			< 0.001			0.359
A-B	Reference			Reference		
C	1.81	1.34-2.43		1.25	0.87-1.81	
D-E	2.01	1.46-2.90		1.09	0.68-1.74	
Domestic crowding (> 2 people/bedroom)			0.125			0.817
No	Reference			Reference		
Yes	1.19	0.95-1.49		0.97	0.75-1.25	
Maternal age (years)			0.562			0,058
< 20	1.24	0.83-1.87		1.63	1.09-2.43	
20-34	1.16	0.83-1.61		1.41	0.98-2.03	
≥ 35	Reference			Reference		
Maternal education (years)			< 0.001			0.085
0-8	2.33	1.72-3.16		1.57	1.05-2.35	
9-11	1.79	1.30-2.45		1.38	0.96-1.99	
≥ 12	Reference			Reference		
Paternal education (years)			< 0.001			0.083
0-8	2.51	1.78-3.54		1.61	1.05-2.47	
9-11	1.79	1.24-2.60		1.37	0.91-2.05	
≥ 12	Reference			Reference		
Maternal skin color			0.081			0.498
White	Reference			Reference		
Mixed-race/Black/Other	1.23	0.97-1.54		0.91	0.71-1.18	
Parity			< 0.001			0.001
1	Reference			Reference		
2	1.48	1.14-1.92		1.34	1.02-1.75	
≥ 3	2.19	1.68-2.85		1.75	1.31-2.34	
Level 2						
Maternal smoking during pregnancy			0.005			0.573
No	Reference			Reference		
Yes	1.45	1.12-1.88		1.09	0.81-1.46	
Level 3						
Sex			< 0.001			< 0.001
Male	1.49	1.20-1.87		1.56	1.24-1.96	
Female	Reference			Reference		
Prematurity			0.011			0.034
No	Reference			Reference		
Yes	1.42	1.08-1.87		1.39	1.05-1.83	
Low birth weight (< 2,500g)			0.185			0.713
No	Reference			Reference		
Yes	1.25	0.90-1.76		1.07	0.74-1.54	

95%CI: 95% confidence interval; PR: prevalence ratio.

### Pneumonia in the second year of life

Table 3 shows the factors associated with pneumonia in the second year of life. Children from adolescent mothers were almost twice as likely to have pneumonia than those from mothers aged ≥ 35 years (PR = 1.98; 95%CI: 1.20-3.28). Children from mothers who had ≥ 3 or 2 children showed a 61% (PR = 1.61; 95%CI: 1.11-2.34) and 55% increased risk of pneumonia, respectively (PR = 1.55; 95%CI: 1.14-2.10), than firstborns. Children who were born prematurely were 49% more likely to have pneumonia than those who were born at term (PR = 1.49; 95%CI: 1.08-2.06). Children who attended daycare centers showed a higher prevalence at 12 months (PR = 1.66; 95%CI: 1.17-2.36). Lower maternal and paternal education, maternal smoking during pregnancy and at 12 months, lack of exclusive breastfeeding at three months, and > 3 people in the household at 12 months increased the prevalence in unadjusted analysis (which lost its statistical significance after the addition of confounders).

**Table 3:** Factors associated with the prevalence of pneumonia at the second year of life. The 2015 Pelotas (Brazil) birth cohort.

Variables	History of pneumonia					
	Unadjusted			Adjusted		
	PR	95%CI	p-value	PR	95%CI	p-value
Level 1						
Family socioeconomic status			0.163			0.873
A-B	Reference			Reference		
C	1.29	0.95-1.75		0.97	0.67-1.40	
D-E	1.38	0.96-1.99		0.89	0.55-1.44	
Domestic crowding (> 2 people/bedroom)			0.237			0.540
No	Reference			Reference		
Yes	1.17	0.90-1.51		1.09	0.82-1.44	
Maternal age (years)			0.183			0.015
< 20	1.46	0.93-2.29		1.98	1.20-3.28	
20-34	1.12	0.76-1.63		1.24	0.83-1.85	
≥ 35	Reference			Reference		
Maternal education (years)			0.024			0.978
0-8	1.54	1.12-2.12		1.01	0.64-1.59	
9-11	1.23	0.89-1.72		0.98	0.68-1.41	
≥ 12	Reference			Reference		
Paternal education (years)			0.004			0.181
0-8	1.69	1.20-2.38		1.34	0.93-1.92	
9-11	1.21	0.82-1.76		1.06	0.72-1.56	
≥ 12	Reference			Reference		
Maternal skin color			0.617			0.617
White	Reference			Reference		
Mixed-race/Black/Other	1.07	0.82-1.40		0.93	0.69-1.24	
Parity			0.013			0.007
1	Reference			Reference		
2	1.41	1.06-1.87		1.55	1.14-2.10	
≥ 3	1.53	1.11-2.10		1.61	1.11-2.34	
Level 2						
Maternal smoking during pregnancy			0.027			0.410
No	Reference			Reference		
Yes	1.40	1.04-1.90		1.15	0.83-1.58	
Level 3						
Sex			0.883			0.967
Male	1.02	0.79-1.31		0.99	0.77-1.28	
Female	Reference			Reference		
Prematurity			0.010			0.015
No	Reference			Reference		
Yes	1.49	1.10-2.02		1.49	1.08-2.06	
Low birth weight (< 2.500g)			0.300			0.734
No	Reference			Reference		
Yes	1.22	0.83-1.81		0.93	0.59-1.44	
Level 4						
Exclusive breastfeeding at 3 months			0.036			0.166
No	1.32	1.01-1.70		1.21	0.92-1.58	
Yes	Reference					
Level 5						
Maternal smoking at 12 months			0.014			0.511
No	Reference			Reference		
Yes	1.45	1.08-1.94		1.12	0.80-1.58	
Breastfeeding at 12 months			0.053			0.264
No	1.29	0.99-1.67		1.21	0.87-1.68	
Yes	Reference			Reference		
Number of people in the household at 12 months			0.015			0.475
≤ 3	Reference			Reference		
> 3	1.40	1.07-1.84		1.13	0.81-1.57	
Attendance to child daycare center at 12 months			0.025			0.005
No	Reference			Reference		
Yes	1.47	1.05-2.06		1.66	1.17-2.36	
Complete pneumococcal vaccination at 12 months			0.303			0.674
No	1.34	0.77-2.35		1.14	0.62-2.07	
Yes	Reference			Reference		

95%CI: 95% confidence interval; PR: prevalence ratio.

### Pneumonia in the third and fourth years of life

In adjusted analysis, none of the independent variables were associated with the prevalence of pneumonia at the third and fourth years of life. In the unadjusted analyses, children who were not breastfed at 24 months showed an increase of 38% in their probability of contracting pneumonia than those who were breastfed (PR = 1.38; 95%CI: 1.03-1.84), but this association disappeared in the adjusted analysis (PR = 1.32; 95%CI: 0.97-1.80) (Table 4).

**Table 4:** Factors associated with prevalence of pneumonia at third and fourth years of life. The 2015 Pelotas (Brazil) birth cohort.

Variables	History of pneumonia					
	Unadjusted			Adjusted		
	PR	95%CI	p-value	PR	95%CI	p-value
Level 1						
Family socioeconomic status			0.606			0.281
A-B	Reference			Reference		
C	0.88	0.67-1.14		0.82	0.62-1.07	
D-E	0.90	0.64-1.26		0.78	0.55-1.13	
Domestic crowding (> 2 people/bedroom)			0.497			
No	Reference			Reference		0.631
Yes	0.92	0.73-1.17		0.94	0.73-1.21	
Maternal age (years)			0.548			0.445
< 20	0.96	0.64-1.45		1.13	0.74-1.72	
20-34	0.86	0.62-1.18		0.91	0.67-1.25	
≥ 35	Reference			Reference		
Maternal education (years)			0.885			0.884
0-8	0.94	0.70-1.25		0.91	0.62-1.33	
9-11	0.94	0.71-1.25		0.95	0.69-1.30	
≥ 12	Reference			Reference		
Paternal education (years)			0.718			0.848
0-8	0.92	0.69-1.23		0.99	0.69-1.44	
9-11	0.89	0.64-1.20		0.92	0.66-1.29	
≥ 12	Reference			Reference		
Maternal skin color			0.938			0.871
White	Reference			Reference		
Mixed-race/Black/Other	1.01	0.78-1.30		0.98	0.73-1.30	
Parity			0.218			0.148
1	Reference			Reference		
2	1.16	0.89-1.51		1.18	0.91-1.55	
≥ 3	1.29	0.96-1.74		1.34	0.99-1.82	
Level 2						
Maternal smoking during pregnancy			0.444			0.253
No	Reference			Reference		
Yes	0.88	0.63-1.22		0.82	0.59-1.15	
Level 3						
Sex			0.226			0.222
Male	0.87	0.69-1.09		0.87	0.69-1.09	
Female	Reference			Reference		
Prematurity			0.330			0.317
No	Reference			Reference		
Yes	1.17	0.86-1.56		1.17	0.86-1.59	
Low birth weight (< 2,500g)			0.468			0.824
No	Reference			Reference		
Yes	1.15	0.79-1.66		1.05	0.68-1.62	
Level 4						
Exclusive breastfeeding at three months			0.133			0.352
No	1.20	0.95-1.52		1.13	0.87-1.46	
Yes	Reference			Reference		
Level 5						
Maternal smoking at 24 months			0.205			0.175
No	Reference			Reference		
Yes	0.85	0.67-1.09		0.80	0.59-1.10	
Breastfeeding at 24 months			0.028			0.081
No	1.38	1.03-1.84		1.32	0.97-1.80	
Yes	Reference			Reference		
Number of people in the household at 24 months			0.848			0.481
≤ 3	Reference			Reference		
> 3	1.02	0.81-1.30		0.90	0.67-1.21	
Attendance to child daycare center at 24 months			0.310			0.277
No	Reference			Reference		
Yes	1.13	0.89-1.45		1.15	0.89-1.48	
Complete pneumococcal vaccination at 24 months			0.145			0.432
No	1.25	0.93-1.68		1.13	0.83-1.55	
Yes	Reference					

95%CI: 95% confidence interval; PR: prevalence ratio.

### Pneumonia in the fifth and sixth years of life

Children of mothers with ≥ 3 or 2 children had a higher probability of having pneumonia than firstborns in the unadjusted and adjusted analyses (Table 5). The strength of the association between parity and the period prevalence increased when adjusting for confounders. Children from mothers with ≥ 3 or 2 children had a twice as high probability of contracting pneumonia at this period (PR = 2.00; 95%CI: 1.13-3.49; and PR = 1.95; 95%CI: 1.20-3.16, respectively). The remaining characteristics showed no association with the prevalence of pneumonia in the adjusted or unadjusted analyses.

**Table 5:** Factors associated with prevalence of pneumonia at the fifth and sixth years of life. The 2015 Pelotas (Brazil) birth cohort.

Variables	History of pneumonia					
	Unadjusted			Adjusted		
	PR	95%CI	p-value	PR	95%CI	p-value
Level 1						
Family socioeconomic status			0.997			0.961
A-B	Reference			Reference		
C	1.00	0.67-1.49		0.94	0.61-1.46	
D-E	1.02	0.62-1.67		0.97	0.50-1.87	
Domestic crowding (> 2 people/bedroom)			0.054			0.203
No	Reference			Reference		
Yes	0.72	0.51-1.00		0.78	0.54-1.14	
Maternal age (years)			0.817			0.733
< 20	0.88	0.46-1.67		1.13	0.57-2.28	
20-34	1.04	0.64-1.68		1.21	0.74-1.98	
≥ 35	Reference			Reference		
Maternal education (years)			0.727			0.728
0-8	1.18	0.77-1.80		1.22	0.74-2.01	
9-11	1.14	0.74-1.75		1.15	0.74-1.78	
≥ 12	Reference			Reference		
Paternal education (years)			0.736			0.747
0-8	1.00	0.65-1.54		0.98	0.63-1.54	
9-11	0.86	0.53-1.37		0.85	0.53-1.37	
≥ 12	Reference			Reference		
Maternal skin color			0.863			0.960
White	Reference			Reference		
Mixed-race/Black/Other	0.97	0.67-1.40		1.01	0.66-1.54	
Parity			0.028			0.016
1	Reference			Reference		
2	1.65	1.13-2.42		1.95	1.20-3.16	
≥ 3	1.47	0.94-2.30		2.00	1.13-3.49	
Level 2						
Maternal smoking during pregnancy			0.373			0.519
No	Reference			Reference		
Yes	1.21	0.79-1.86		1.15	0.75-1.78	
Level 3						
Sex			0.407			0.259
Male	0.87	0.62-1.21		0.82	0.58-1.15	
Female	Reference			Reference		
Prematurity			0.700			0.949
No	Reference			Reference		
Yes	1.09	0.69-1.73		0.98	0.54-1.78	
Low birth weight (< 2,500g)			0.325			0.333
No	Reference			Reference		
Yes	1.29	0.77-2.16		1.30	0.76-2.20	
Level 4						
Exclusive breastfeeding at three months			0.098			0.118
No	1.34	0.95-1.90		1.32	0.92-1.89	
Yes	Reference			Reference		
Level 5						
Maternal smoking at 48 months			0.263			0.352
No	Reference			Reference		
Yes	1.26	0.84-1.91		1.23	0.79-1.91	
Breastfeeding at 48 months			0.907			0.608
No	0.96	0.47-1.94		0.83	0.41-1.69	
Yes	Reference			Reference		
Number of people in the household at 48 months			0.881			0.170
≤ 3	Reference			Reference		
> 3	1.03	0.73-1.45		0.72	0.46-1.15	
Attendance to child daycare center at 48 months			0.926			0.769
No	Reference			Reference		
Yes	0.98	0.70-1.38		1.05	0.72-1.54	
Complete pneumococcal vaccination at 48 months			0.180			0.164
No	1.54	0.82-2.89		1.57	0.83-2.97	
Yes	Reference			Reference		

95%CI: 95% confidence interval; PR: prevalence ratio.

### Pneumonia from birth up to six years of life

For the entire period, prevalence was 1.62 and 1.38 times higher in children from mothers with ≥ 3 or 2 previous children, respectively, than in firstborns (PR = 1.62; 95%CI: 1.33-1.96, PR = 1.38; 95%CI: 1.16-1.64) (Table 6). The occurrence of pneumonia was higher in premature children (PR = 1.34; 95%CI: 1.11-1.61). Maternal and paternal education, which increased the probability of pneumonia in the unadjusted analysis, lost its statistical significance after the addition of confounders.

**Table 6:** Factors associated with prevalence of pneumonia from birth to six years of life. The 2015 Pelotas (Brazil) birth cohort.

Variables	History of pneumonia					
	Unadjusted			Adjusted		
	PR	95%CI	p-value	PR	95%CI	p-value
Level 1						
Family socioeconomic status			0.106			0.633
A-B	Reference			Reference		
C	1.72	0.98-1.40		1.07	0.87-1.32	
D-E	1.24	1.00-1.54		0.99	0.74-1.31	
Domestic crowding (> 2 people/bedroom)			0.720			0.787
No	Reference			Reference		
Yes	1.03	0.89-1.19		0.98	0.83-1.15	
Maternal age (years)			0.915			0.051
< 20	1.06	0.81-1.39		1.43	1.07-1.90	
20-34	1.04	0.84-1.28		1.17	0.95-1.45	
≥ 35	Reference			Reference		
Maternal education (years)			0.009			0.686
0-8	1.32	1.10-1.59		1.08	0.83-1.41	
9-11	1.12	0.93-1.35		0.99	0.79-1.23	
≥ 12	Reference			Reference		
Paternal education (years)			0.027			0.441
0-8	1.27	1.04-1.54		1.11	0.90-1.37	
9-11	1.07	0.86-1.32		1.00	0.81-1.24	
≥ 12	Reference			Reference		
Maternal skin color			0.217			0.686
White	Reference			Reference		
Mixed-race/Black/Other	1.10	0.94-1.29		0.96	0.81-1.15	
Parity			< 0.000			< 0.000
1	Reference			Reference		
2	1.28	1.09-1.52		1.38	1.16-1.64	
≥ 3	1.51	1.25-1.81		1.62	1.33-1.96	
Level 2						
Maternal smoking during pregnancy			0.190			0.869
No	Reference			Reference		
Yes	1.13	0.94-1.37		1.02	0.84-1.23	
Level 3						
Sex			0.203			0.219
Male	1.10	0.95-1.27		1.09	0.95-1.27	
Female	Reference			Reference		
Prematurity			0.003			0.005
No	Reference			Reference		
Yes	1.32	1.10-1.59		1.34	1.11-1.61	
Low birth weight (< 2,500g)			0.265			0.706
No	Reference			Reference		
Yes	1.14	0.90-1.44		0.95	0.73-1.24	

95%CI: 95% confidence interval; PR: prevalence ratio.

### Reoccurrence of pneumonia from birth up to 6 years of life

Table 7 describes the factors associated with more than one episode of pneumonia from birth to six years of life. Children of adolescent mothers (PR = 2.58; 95%CI: 1.55-4.29) and of mothers with higher parity showed a higher probability of reoccurrence. Children of mothers who had ≥ 3 or 2 children had a 132% (PR = 2.32; 95%CI: 1.63-3.30) and 71% (PR = 1.71; 95%CI: 1.23-2.37) higher probability of the same outcome than firstborns, respectively. Maternal and paternal education lost its statistical significance after adjusting for confounders.

**Table 7:** Factors associated with recurrence of pneumonia from birth to six years of life. The 2015 Pelotas (Brazil) birth cohort.

Variables	History of pneumonia					
	Unadjusted			Adjusted		
	PR	95%CI	p-value	PR	95%CI	p-value
Level 1						
Family socioeconomic status			0.805			0.368
A-B	Reference			Reference		
C	1.08	0.78-1.49		0.82	0.56-1.20	
D-E	1.14	0.76-1.70		0.68	0.40-1.16	
Domestic crowding (> 2 people/bedroom)			0.747			0.994
No	Reference			Reference		
Yes	1.05	0.79-1.38		1.00	0.73-1.37	
Maternal age (years)			0.143			0.001
< 20	1.56	0.95-2.57		2.58	1.55-4.29	
20-34	1.16	0.76-1.77		1.40	0.92-2.13	
≥ 35	Reference			Reference		
Maternal education (years)			0.041			0.342
0-8	1.53	1.07-2.20		1.11	0.68-1.82	
9-11	1.52	1.06-2.18		1.30	0.88-1.91	
≥ 12	Reference			Reference		
Paternal education (years)			0.030			0.413
0-8	1.55	1.07-2.26		1.39	0.89-2.18	
9-11	1.13	0.74-1.70		1.07	0.70-1.63	
≥ 12	Reference			Reference		
Maternal skin color			0.960			0.450
White	Reference			Reference		
Mixed-race/Black/Other	0.99	0.74-1.34		0.89	0.66-1.20	
Parity			0.003			0.000
1	Reference			Reference		
2	1.40	1.02-1.92		1.71	1.23-2.37	
≥ 3	1.79	1.28-2.51		2.32	1.63-3.30	
Level 2						
Maternal smoking during pregnancy			0.112			0.576
No	Reference			Reference		
Yes	1.32	0.94-1.85		1.10	0.78-1.56	
Level 3						
Sex			0.964			0.986
Male	1.01	0.77-1.32		1.00	0.76-1.31	
Female	Reference			Reference		
Prematurity			0.215			0.685
No	Reference			Reference		
Yes	1.25	0.88-1.78		1.09	0.73-1.63	
Low birth weight (< 2,500g)			0.123			0.273
No	Reference			Reference		
Yes	1.37	0.92-2.06		1.27	0.85-1.81	

95%CI: 95% confidence interval; PR: prevalence ratio.

## Discussion

This study is one of the first, if not the only one in Brazil, to describe the prevalence and factors associated with pneumonia from age 0 to 6 years in a population-based sample after the introduction of the pneumococcal vaccine in the Brazilian PNI. The prevalence of at least one episode of pneumonia from 0 to 6 years in our study (16.7%) was similar to what was found in India in 2015 (16.3%) ^18^, whereas preschoolers in China showed a higher prevalence in 2019: 26.4% ^19^. In a systematic review and meta-analysis with studies carried out in East Africa and published from 2000 to 2019, the combined prevalence in children < 5 years totaled 34% (95%CI: 23.8-44.21) ^20^.

In our study, except in the third and fourth years of life, higher maternal parity offered the strongest and most consistent factor associated with a higher incidence of pneumonia. All follow-ups showed a similar strength of this association. Previous studies have also reported the importance of parity as a risk factor for childhood pneumonia, suggesting that the circulation of respiratory pathogens such as viruses and bacteria may be more intense in families with more children, increasing the risk of infections for younger children, especially in inadequate housing conditions ^3^ ^,^ ^21^ ^,^ ^22^ ^,^ ^23^ ^,^ ^24^.

From the child’s individual conditions, we found an association with sex and gestational age. The male sex was associated with a greater occurrence of pneumonia in the first year of life, and prematurity was associated with greater risk of pneumonia in the first and second years. Several studies suggest a greater occurrence of pneumonia in boys ^3^ ^,^ ^24^ ^,^ ^25^ ^,^ ^26^ ^,^ ^27^. Men show greater susceptibility to most types of respiratory tract infections in all age groups (adults and children) than women ^28^. Anatomical differences (narrower peripheral airways during the first years of life), parental style, and behavioral and socioeconomic differences may cause such findings ^28^. Regarding the association with gestational age, preterm delivery configures a risk factor for pneumonia ^1^ ^,^ ^21^ ^,^ ^29^ ^,^ ^30^, in which the immaturity of children’s immune, respiratory, and pulmonary systems may constitute a possible mechanism ^21^ ^,^ ^30^.

We were unable to detect the expected protective effect of the pneumococcal vaccine on the occurrence or reoccurrence of pneumonia ^31^ ^,^ ^32^ ^,^ ^33^ ^,^ ^34^ ^,^ ^35^. The vaccination coverage in our study was almost universal, causing a small proportion of unvaccinated children to give rise to statistical significance in prevalence differences. A study carried out in Australia showed that the protective effect of PCV13 in preventing hospitalization for pneumonia in children aged under 12 months was higher than the effect of PCV10 ^36^. Thus, the prevalence of pneumonia in our sample would possibly be lower if the PCV13 was available in our setting. A study carried out by the Brazilian Ministry of Health in 2023 found no justification for the cost-benefit of replacing PCV10 by PCV13 at that moment ^37^. Additionally, we have no information on whether the child received the pneumococcal vaccine in the public or private sector. Nonetheless, Pelotas had few private clinics offering PCV13 in 2015. Furthermore, a significant portion of the population in Pelotas depends on the SUS (as most Brazilian municipalities). According to data from the Brazilian Institute of Geography and Statistics (IBGE, acronym in Portuguese, an estimated 70% to 80% of the population of Pelotas exclusively uses the SUS for medical care and access to health services, such as consultations, exams, medications, and hospitalizations ^38^.

Previous studies have shown that low birth weight was associated with 3.2 times greater odds of severe pneumonia in low- and middle-income countries and 1.8 times greater odds in high-income countries ^3^. Similarly, lack of exclusive breastfeeding in the first four months of life increases the likelihood of severe pneumonia by 2.7 times in low- and middle-income countries and by 1.3 times in high-income countries ^3^ ^,^ ^7^. Likewise, household crowding configures a risk factor in low-, middle- and high-income countries, with probability ratios from 1.9 to 2.3, as smoking indoors, which increases the chances of pneumonia by 1.6 times ^3^. Nonetheless, none of these risk factors were associated with a higher occurrence of pneumonia in our study. Although the improvements in socioeconomic conditions in the population of Pelotas in recent years ^39^ may have contributed to reducing cases and deaths from respiratory infections in childhood ^40^, the high efficacy of the vaccine ^41^ in protecting both vaccinees (by direct protection) and the broader population (by indirect or herd protection) may have been associated with its high population coverage, attenuating the strength of the association of those factors with the risk of developing pneumonia in childhood.

Regarding family socioeconomic and environmental characteristics, we found an association between child daycare center attendance and a higher probability of pneumonia in the second year of life. Attendance at daycare centers has been reported to predispose children to pneumonia, which is normally attributed to an increased chance of contagion with the pathogens in respiratory tract infections by contact with other children ^3^ ^,^ ^21^ ^,^ ^22^.

Children of adolescent mothers had a higher risk of pneumonia in their second year of life. Consistent with our finding, a previous study ^22^ has shown that the risk of hospitalization due to pneumonia in children of teenage mothers was twice as high as that of children from mothers aged 20-34 years. Other recognized risk factors, such as family low socioeconomic level, lower maternal and paternal education, and maternal non-white skin color, showed no association with a higher prevalence of pneumonia in our study.

The strengths of this study include its prospective design, large sample size, and the low attrition rate in all follow-ups. Prospective cohort designs provide accurate information about exposures as they minimize recall biases. However, this study also has some limitations. It defined pneumonia based on reports from mothers/guardians. Because the children were not examined and there was no medical documentation available, this could have led to information bias. To minimize this limitation, this research asked mothers/guardians whether physicians had performed their children’s diagnosis, only considering those with a positive answer to this question as parents of pneumonia cases. Other studies have shown that malnutrition configures a risk factor for pneumonia and the association between low weight for age and severe respiratory infections in low- and middle-income countries ^7^ ^,^ ^25^. We did not include this variable in our study because, as recent results suggest, the 2015 Pelotas birth cohort is experiencing a nutritional transition as its prevalence of stunting decreased by 53% in comparison to the 1982 Pelotas birth cohort (from 8.3% to 3.9%). The prevalence of low weight for age remains stable at low levels (1.8% in 1982 and 1.7% in 2015), whereas excessive weight increased by 88% (6.5% to 12.2%) ^42^. Another limitation in this study refers to its lack of information on the etiology of pneumonia and other agents, such as viruses, that may have been involved in the reported cases. Also, for those without vaccination cards at the moment of the interview, this study collected the information on immunization with the pneumococcal vaccine from mothers’ verbal reports, which may have given rise to information bias.

## Conclusion

In summary, we found that, in a population with high PCV10 vaccination coverage, almost one in five children aged 6 years had a positive history of pneumonia, and most cases occurred in their first year of life. Greater maternal parity configured the strongest and most consistent factor associated with a higher prevalence of pneumonia. Prematurity was related to pneumonia in the first two years of life and the male sex in the first year only. Adolescent mothers increased the likelihood of pneumonia in the second year of life and the likelihood of pneumonia reoccurrence. Attendance to child daycare centers was related to pneumonia only in the second year of life. Such findings indicate that these factors should be kept in mind when searching for children at increased risk of pneumonia.

## Data Availability

The research data are available upon request to the corresponding author.
